# From Expert to Elite? — Research on Top Archer’s EEG Network Topology

**DOI:** 10.3389/fnhum.2022.759330

**Published:** 2022-02-25

**Authors:** Feng Gu, Anmin Gong, Yi Qu, Aiyong Bao, Jin Wu, Changhao Jiang, Yunfa Fu

**Affiliations:** ^1^School of Information Engineering, Engineering University of People’s Armed Police, Xi’an, China; ^2^School of Military Basic Education, Engineering University of People’s Armed Police, Xi’an, China; ^3^Department of Physical Education, Beijing City University, Beijing, China; ^4^Key Laboratory of Sports Performance Evaluation and Technical Analysis, Capital Institute of Physical Education, Beijing, China; ^5^School of Automation and Information Engineering, Kunming University of Science and Technology, Kunming, China

**Keywords:** electroencephalography, elite archer, weighted phase lag index (WPLI), function coupling change rate, brain network topology

## Abstract

It is not only difficult to be a sports expert but also difficult to grow from a sports expert to a sports elite. Professional athletes are often concerned about the differences between an expert and an elite and how to eventually become an elite athlete. To explore the differences in brain neural mechanism between experts and elites in the process of motor behavior and reveal the internal connection between motor performance and brain activity, we collected and analyzed the electroencephalography (EEG) findings of 14 national archers and 14 provincial archers during aiming and resting states and constructed the EEG brain network of the two archer groups based on weighted phase lag index; the graph theory was used to analyze and compare the network characteristics *via* local network and global network topologies. The results showed that compared with the expert archers, the elite archers had stronger functional coupling in beta1 and beta2 bands, and the difference was evident in the frontal and central regions; in terms of global characteristics of brain network topology, the average clustering coefficient and global efficiency of elite archers were significantly higher than that of expert archers, and the eigenvector centrality of expert archers was higher; for local characteristics, elite archers had higher local efficient; and the brain network characteristics of expert archers showed a strong correlation with archery performance. This suggests that compared with expert archers, elite archers showed stronger functional coupling, higher integration efficiency of global and local information, and more independent performance in the archery process. These findings reveal the differences in brain electrical network topologies between elite and expert archers in the archery preparation stage, which is expected to provide theoretical reference for further training and promotion of professional athletes.

## HIGHLIGHTS

- In the beta1 and beta2 frequency bands near the shooting time, the information interaction between the frontal and central regions of the elite archers’ brains is closer than that of the expert archers.
- The clustering coefficient, global and local efficiency of elite archers is significantly higher than that of expert archers in beta1 and beta2 frequency bands, but the characteristic path length is opposite.
- Compared with expert archers, elite archers showed fewer brain regions significantly correlated with archery performance in terms of information interaction and local information integration efficiency.

## Introduction

Exploring the physiological mechanism underlying exercise behavior execution is an important research topic in sports science (Gong et al., 2020). Previous researchers demonstrated a close relationship between the neural activities of athletes during exercise and their sports surfaces. By examining the neural markers closely related to sports performance, researchers can more accurately understand the neural activity rules underlying sports behaviors (Park et al., 2015). Electroencephalography (EEG) is a classic method of recording electrical activity generated by brain neurons on the scalp surface (da Silva, 2009) and has been widely used in the treatment of neurological diseases, emotion recognition, exercise, and other fields of research (Hatfield et al., 1984; Tao et al., 2005; Zhang et al., 2019). Fine motion (e.g., archery and shooting) is an area of intense interest to researchers. By measuring EEG signals of athletes at different competitive levels and analyzing various types of EEG characteristics (amplitude, power, functional coupling, etc.), researchers explored the relationship between performance and neural mechanisms during exercise. Salazar et al. (1990) found that the higher the alpha and beta1 wave power of the left temporal region of athletes, the worse was their archery performance during the shooting stage (Salazar et al., 1990). Loze et al. compared the changes in the alpha wave power of the occipital region corresponding to the best and worst shooting performance and showed that the alpha wave power increased in the best shooting performance. In contrast, the worst shooting performance showed a downward trend (Loze et al., 2001). Gong et al. analyzed the brain activity of 40 skilled shooters using the phase-locking value (PLV)-based functional connection values and complex networks. They found that shooters with higher performance showed lower functional coupling and higher global and local information integration efficiency during firing (Gong et al., 2018). Zhang et al. (2021) compared the EEG and ECG characteristics of 11 national-level archers under competitive shooting and non-competitive shooting tasks. They found that the theta power in occipital regions, alpha power in frontal-central and left occipital regions, and beta power in frontal and mid-occipital regions in the competitive state were significantly higher than those in the corresponding regions in the non-competitive state (Zhang et al., 2021).

In the history of sports science, exploring the physiological differences among athletes at different competitive levels has always been a topic of interest (Doppelmayr et al., 2008; Luchsinger et al., 2016; Lu et al., 2020). From the perspective of “neural efficiency,” several studies have demonstrated that professional athletes can perform better with less energy expenditure on neural activity (Neubauer and Fink, 2009; Callan and Naito, 2014; Chang, 2014). This is manifested as reduced neural activity in specific brain regions, thereby making the brain less controlled and more automated (Debarnot et al., 2014). For example, Haufler et al. (2000) compared the EEG findings of experienced shooters and novices during aiming and found that experienced shooters showed lower activation in all brain regions than novices, with significant differences in activation in the left central temporoparietal region. Del Percio et al. found that the range of event-related desynchronization (ERD) in the alpha1 and alpha2 bands of the whole brain of experts was lower than that of beginners during the aiming stage of shooting. In addition, the range of event-related synchronization (ERS) in the alpha2 frequency band is significantly positively correlated with the shooting performance (Del Percio and Babiloni, 2009). In a subsequent study, they discovered that the amplitude of event-related coherence in the hemispheres and between the hemispheres (parietal–temporal and parietal–occipital regions) of professional shooting athletes was more stable in the multiple frequency bands (Del Percio et al., 2011b). In terms of EEG functional coupling characteristics, the coherence in the brain regions of professional athletes was significantly lower than in those of novice athletes (Deeny et al., 2003, 2009). In the study of the archery process, the visual association cortex of the left occipital lobe and anterior cingulate was more activated in expert archers, whereas the frontal region was more activated in novice archers (Kim et al., 2008). Neurofeedback was regarded as an effective method to train athletes to improve their performance in sports psychology. A study comparing the arousal levels of archers before and after neurofeedback intervention found statistically significant changes in the SMR/theta ratio of archers who had received neurofeedback training after the competition (Paul et al., 2012).

Previous research on the EEG neural mechanism of athletes with different skill levels in fine motion mainly focused on comparing experts and novices. However, few studies have discussed the differences in the brain function of professional athletes from the perspective of comparing experts and elites. Archery is characterized by fine motion and a set of action sequences such as holding the bow and drawing, anchoring, loading, aiming, sustaining release, and follow-through of the arrow (Lee, 2005). Perfect archery performance depends on stable posture as well as coordinated movement and requires sustained concentration and good psychological quality (Lee, 2009; Sarro et al., 2021). After long-term professional training, most professional archery athletes master similar archery skills; however, owing to psychological factors and neurological reasons, it is difficult to break through the bottleneck and grow into top archery athletes (Zhou and Liu, 2010). Psychology is the reflection of the brain’s activities; the mental activity of an archer during archery preparation is usually accompanied by changes in the brain’s internal nerves; the brain performs complex processes to recognize stimuli; select and plan responses; make decisions; prepare; and execute actions (Gladwin et al., 2006; Cañal-Bruland et al., 2010). The importance of highly concentrated attention for excellent archery performance has been reported in the cognitive psychology literature (Bu et al., 2019; Lu et al., 2021); however, currently, for skilled archers with different competitive levels but with professional training, there remains a lack of understanding regarding the rhythm, the connection between brain regions, the network topology, and whether these characteristics are closely associated with archery performance. What are the differences between expert shooters in terms of brain function? How to select athletes with the potential to become elite archers based on these brain characteristics? Furthermore, formulating a scientific training plan based on these brain characteristics to enhance the competitive level of expert archers is a concern for coaches and professional athletes.

To further examine the neural mechanism of archers with different competitive levels during aiming, this experiment adopted the most popular brain network analysis to reveal the differences in functional connections and network characteristics between elite and expert athletes using a data-driven method. We collected EEG findings of 14 national archery athletes (elite group) and 14 provincial archery athletes (expert group) during the targeting stage. Using weighted phase lag index (WPLI) algorithm to evaluate the function of the EEG findings in different band connectivity, this phase-based method reduces the volume conduction effect to a certain extent and prevents the mixing of phase and amplitude factors (Vinck et al., 2011). Unlike previous research, we proposed the characteristics of the event-related functional coupling change rate based on WPLI to describe the significant changes of brain connectivity during aiming. Then, we employed graph theory to analyze the brain network based on WPLI through exploration of the global and local topologies of the network analyzed in subjects when performing archery aiming tasks (Fu et al., 2020) and the neural characteristics correlated with archery performance to better understand the changing EEG characteristics of professional archers during the archery preparation process (Wang et al., 2010). In this study, it was assumed that there were significant differences in EEG functional coupling characteristics and brain network topologies between elite and expert archers in the archery preparation stage, and there was also a significantly close relationship between these brain network characteristics and the archery performance. Our study had certain practical guiding significance, which not only revealed differences between the neural mechanisms of elite and expert archers, but also provided key physiological bases for the evaluation of the training state of archers, and was expected to lay a theoretical foundation for the improvement of archery performance through neurofeedback training.

## Materials and Methods

### Subjects

This study included a total of 31 experimental subjects who were categorized into elite and expert groups. The elite group comprised 16 archers from the Chinese national archery team (10 men, 6 women, age: 23 ± 5 years), and their average training age was 8.1 years. The expert group comprised 15 archers from the Beijing archery team (9 men, 6 women, age: 23 ± 5 years), and their average training age was 6.1 years. We employed the one-way ANOVA to examine the age and sex differences between the groups and found no significant difference between the two archer groups in terms of age and sex (age: *p* > 0.05, sex: *p* > 0.05).

Elite archers are classified as masters or international masters who have participated in international competitions such as the Olympics, Asian Games, and World Cup, and their average FITA score is 58.5. On the other hand, expert archers are the national first-class or second-class athletes who have participated in national championships but have not participated in international competitions. Please refer to Supplementary Appendix 1 for details of these subjects.

All subjects participated in gunnery training at least 4 days a week and 6 h a day before the experiment. All the subjects were right-handed, with their left hand holding the bow and their right hand hanging the string. Among them, three participants in the elite group and two in the expert group had vision correction. After correction, all subjects had normal visual acuity. The subjects had no major head injury, craniotomy, and mental disease and had good physical function. They did not consume alcohol, coffee, tea, and other stimulant drinks 24 h before the experiment and did not consume any neurogenic drugs that could interfere with the study results. The experimental site was the outdoor range of the national archery team, and the experiment was conducted under the guidance of professional coaches. The experiment was reviewed by the Ethics Committee of the Capital Institute of Physical Education. All subjects understood the content of the experiment and signed an agreement before participating in the experiment.

### Electroencephalography Acquisition

**FIGURE 1:**
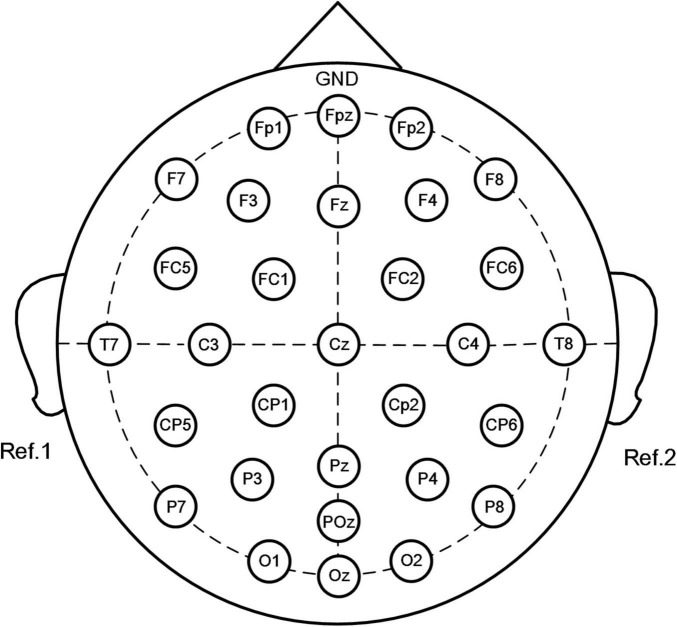
Placement of electrodes using the standard 10–20 system (30 channels: Fp1, Fpz, Fp2, F7, F3, Fz, F4, F8, FC5, FC1, FC2, FC6, T7, C3, Cz, C4, T8, CP5, CP1, CP2, CP6, P7, P3, Pz, P4, P8, POz, O1, Oz, and O2. Forehead: ground, left, and right mastoid processes: references).

Electroencephalography was performed using the SAGA 30 channel EEG amplifier manufactured by TMSI (Netherlands), which is portable and meets the mobility requirements of this experiment. Electrode placement was in accordance with the international 10–20 system, comprising 30 electrodes. The electrode positions were Fp1, Fpz, Fp2, F7, F3, Fz, F4, F8, FC5, FC1, FC2, FC6, T7, C3, Cz, C4, T8, CP5, CP1, CP2, CP6, P7, P3, Pz, P4, P8, POz, O1, Oz, and O2, with a ground electrode placed at the forehead and the reference electrodes placed at the left and right mastoid. Detailed electrode placement is presented in Figure 1, and the sampling frequency was 500 Hz. Before the experiment, the impedance of all electrodes was adjusted to keep it below 5 kΩ, and then the resting state EEG and the EEG of the entire shooting process were collected.

**FIGURE 2:**
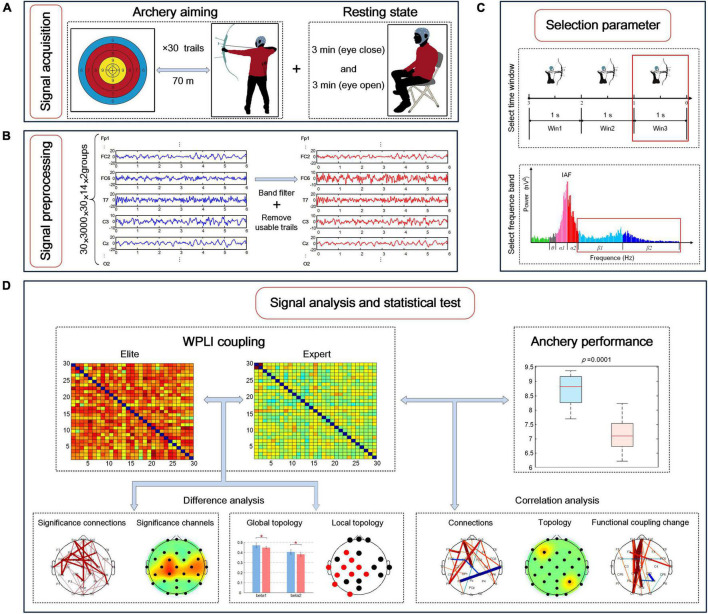
Electroencephalography (EEG) brain network research involving experimental signal acquisition and data analysis process during archery aiming. **(A)** Experimental signal acquisition; **(B)** experimental signal pretreatment process; **(C)** the division of the time window and frequency band during the targeting stage, and the selection of the time window and frequency band with the most significant difference in the functional coupling matrix between the two archer groups; **(D)** the difference analysis of EEG characteristics between the two groups and the correlation analysis and archery performance.

In the experiment, all subjects completed the EEG signal acquisition of the same task. As shown in Figure 2A, the subjects’ resting-state EEG was collected first, with the subjects seated on a soft, comfortable seat and relaxed. During the resting state collection, the subjects were asked to not recall anything deliberately and to keep their eyes closed for 3 min and open for 3 min separately. Next, EEG signals were collected during the entire archery process. The athletes used their familiar bows and arrows to aim at the standard target paper of the international archery competition placed 70 m away. The size of the target paper was 52 cm × 52 cm, the diameter of the 10 rings was 10 cm, the edges of the 10 rings extended outward every time, and the edges of the 10 rings extended 5 cm outward for 9, 8, 7, and 6 rings in sequence. Each time the athlete executed a shooting, the target reporter provided feedback on the shooting result after the firing, and the shooter adjusted the aiming point according to the result. Athletes performed shooting at their own pace, performing a total of 35 shots. The archery shooting time was recorded by automatic infrared sensing, and the time point of firing was marked for evaluating the EEG signal. According to the target paper, the shooting score was recorded as 6–10 points (0 points for missing the target). The archery process of all athletes was independently conducted, and each athlete was not aware of the result of other athletes. Before the experiment, the athletes were told not to bother about the result but to focus on their archery skills.

### Data Preprocessing and Experimental Procedure

The collected EEG signals were transmitted to a computer for offline processing, as shown in Figure 2B. First, a finite impulse response (FIR) filter with an order of 200 was used for bandpass filtering of 0.1–50 Hz for all signals. Next, data were segmented: the EEG signal data from 6 s before the archery of the shooting time were recorded as a trial. Because the neural activity of archers rapidly changes during the aiming stage, the experiment required a high degree of refinement. Therefore, considering the 1 s duration as a data segment, the continuous EEG signal was divided into three-time windows: −3 to −2 s, −2 to −1 s, and −1 to 0 s, defined as Win1, Win2, and Win3, respectively. Next, using the EEGLAB toolbox, Swartz Center for Computational Neuroscience, Institute for Neural Computation, University of California San Diego to visually assess the EEG to be more affected by the artifacts, two athletes of the national archery team and one athlete of the Beijing archery team were removed owing to excessive artifacts. Next, we used independent component analysis (ICA) to remove electrooculogram artifacts, yielding the filtered signals. Finally, a total of 856 available tests were performed for 28 subjects in the two groups during the aiming stage, with an average of 30 tests remaining for each subject (removal rate ≈ 17%).

To reduce individual differences among the subjects, we used the individual alpha frequency (IAF) method for determining the frequency division of different subjects. IAF refers to the frequency band between 8 and 13 Hz (Del Percio et al., 2011b). First, with every 4 s as a segment, and the average power position of the occipital electrodes (O1, O2, and Oz) in the highest frequency band between 8 and 13 Hz (frequency resolution 0.5 Hz) was calculated as the IAF of the subjects in the resting state with eyes closed using the fast Fourier transform (FFT) method. Next, the sub-band frequency of each subject was determined according to IAF: the theta frequency band was defined as IAF-6 to IAF-3 (Hz), alpha1 frequency band was defined as IAF-2 to IAF (Hz), alpha2 frequency band was defined as IAF to IAF+2 (Hz), beta1 frequency band was defined as IAF+3 to 20 (Hz), and beta2 frequency band was defined as 20 to 30 (Hz).

The data analysis process is summarized in Figures 2C,D: first, the frequency bands and time windows with the largest difference were selected for analysis by comparing the difference between the average WPLI connection strength of the elite and expert groups; next, EEG characteristics of the two groups were extracted in the selected frequency band and time window, including the strength of functional coupling, the rate of change of functional coupling as well as global and local brain network topologies; finally, the difference test was conducted on the above characteristics of the two archer groups, and the correlation test was conducted with the archery performance of the two archer groups, and the statistical test results were obtained.

### Electroencephalography Functional Coupling Based on Weighted Phase Lag Index

In this study, we used the WPLI method to calculate the functional coupling between EEG signals. WPLI method is an algorithm to measure the degree of connection by calculating the phase synchronization relationship between signals (Vinck et al., 2011). Compared with the coherence-based functional connection algorithm and other phase functional connection algorithms (such as PLV and PLI algorithm), WPLI indicates the phase lead and lag based on the magnitude of the virtual part of the cross-spectrum, which reduced the volume conduction effect to a certain extent and prevented the impact of the mixing of phase and amplitude. Simultaneously, the statistical power of detecting phase synchronization changes increases. Because of its low computational complexity and solid statistical efficiency in phase detection, this method is also suitable for the functional coupling of real-time monitoring and calculation.

Before WPLI calculation, to obtain the same phase and amplitude of EEG signals of the two archer groups, first, according to the divided frequency band obtained by IAF, FIR bandpass filters of theta, alpha1, alpha2, beta1, and beta2 bands were constructed to perform bandpass filtering on the EEG signals of all subjects in the two groups. Next, the Hilbert transform was applied to the filtered narrowband signals to obtain the two groups of analytical signals of each subject in a specific frequency band. Finally, 10% of the data at both ends of the analytic signals were removed. Based on the results obtained, the WPLI value between the two groups of EEG signals and the two groups of different channels was calculated using the following formula (Vinck et al., 2011):


(1) $${\text{WPLI}}_{\text{xy}}=\frac{\left|\left\langle |ℑ\left({\text{S}}_{\text{xy}}\left(\text{t}\right)\right)|\text{sign}\left(ℑ\left({\text{S}}_{\text{xy}}\left(\text{t}\right)\right)\right)\right\rangle \right|}{\left\langle \left|ℑ\left({\text{S}}_{\text{xy}}\left(\text{t}\right)\right)\right|\right\rangle }$$

The S_xy_(t) represents the cross-spectrum of EEG signals *x*(*t*) and *y*(*t*), ℑ(•) represents the imaginary part, and (•) represents the average value for a given period of time. The value of WPLI ranges from 0 to 1. The higher the value of WPLI, the higher the coupling degree of oscillatory neural activity.

After preprocessing, the EEG data format of each group was 30 × 1,000 × 30 × 14, representing 30 channels, 1,000 data sampling points, 30 trials, and 14 subjects in each group. Next, the WPLI between all possible EEG channels was calculated, and 30 × 30 matrices of the coupling values of the subjects were obtained in each time window and frequency band. Therefore, the data format of each group of functional coupling matrix was 30 × 30 × 5 × 3 × 14, representing 30 × 30 WPLI connection matrix, 5 analyzed frequency bands, three-time windows, and 14 subjects. In addition, to study the connection values of each channel, we obtained the average connection strength of different channels obtained by averaging the row/column direction of the two groups of WPLI coupling matrices (Gong et al., 2019).

To study the changes in the neural mechanism of professional archers before firing, the concept of event-related functional coupling change rate based on WPLI (ErWPLI) was used to describe the changes in functional coupling strength of the two archer groups. The calculation formula is as follows:


(2) $$\text{E}rWPLI\left(t,f\right)=\frac{WPLI\left(t,f\right)-\bar{R\left(f\right)}}{\bar{R\left(f\right)}}$$

Where *WPLI*(*t*, *f*) is the functional coupling strength based on WPLI in a certain time band and *R*(*f*) represents the functional coupling strength of each frequency band at the baseline. In this article, the average WPLI connection strength of the two archer groups in −4 to −3 s before the shooting was considered the baseline, and the change rate of the functional coupling strength of the two archer groups in different frequency bands in three-time windows was calculated. The above-described calculation was performed using the MATLAB R2014a platform.

### Topological Characteristics of Functional Brain Networks During Aiming

In this experiment, the brain network research method based on graph theory was used to analyze the topological characteristics of the brain network of the WPLI connection matrix of the elite and expert groups. The analysis procedure is to transform the functional connection matrix obtained by the WPLI into the adjacency weight matrix, and then using the method of graph theory, different regions of the brain are regarded as nodes, and the connections are regarded as edges, so as to extract all kinds of topological characteristics of the brain networks of each subject in each group at each analysis frequency band (Gong et al., 2018).

The experiment does not set the threshold, and the coupling values between all nodes are retained to form the functional weight brain network. The subsequent brain network topology analysis is also carried out on the basis of the brain network.

In the experiment, the topological characteristics of global and local brain networks are used to analyze. Among the global topological characteristics, we selected the average clustering coefficient, characteristic path length, and analysis efficiency for analysis. The ratio of the clustering coefficient for the characteristic path length represents the small-world characteristic of the network. For local topological characteristics, the eigenvector centrality, average shortest path length, and local efficiency were selected. Please refer to Supplementary Appendix 2 for the formulas of these topological characteristics. The above calculation process was implemented using the Brain Connectivity Toolbox, Vander Bilt Department of Biomedical Engineering, Vanderbilt University (Rubinov et al., 2009).

### Statistical Analysis

We assumed that there are significant differences in WPLI connection strength, functional coupling change rate, as well as global and local topological characteristics of brain networks between the elite and expert archers. To test this hypothesis, we used the Kolmogorov–Smirnov test (K–S test) to evaluate the EEG characteristics of the two groups, and it was found that all characteristics except the functional coupling change rate do not follow a normal distribution (*p* < 0.05) and not all results in the functional coupling change rate exhibit normal distribution. Because not all characteristics follow a normal distribution, we uniformly adopted the nonparametric test to analyze the samples. Then, the Wilcoxon signed-rank test, one of the nonparametric tests, was used to test whether the differences between the two sets of corresponding values were significant (Zalesky et al., 2010; Gong et al., 2020). We set 0.05 and 0.01 as the test level with significant and very significant differences between the two groups of samples, respectively. Finally, the false discovery rate (FDR) method was adopted to was used to correct the statistical results.

In addition, in this study, we assumed that the functional coupling strength, change rate, and network topology characteristics of the two archer groups are closely correlated with the average performance of 30 shots. To test this hypothesis, a cross-subject statistical test was used to analyze the correlation between EEG characteristics and archery performance of each subject. The K–S test was conducted on the average score of each archery athlete in the two groups after 30 shots, and it was found that the average score did not follow a normal distribution (*p* < 0.05). Therefore, Spearman rank correlation analysis was selected for correlation analysis, and the correlation coefficient (*r*) was calculated. Similarly, we also used FDR to correct the *p*-values in results obtained by the Spearman rank test. The statistical analysis part was also performed using the statistical test toolbox of the MATLAB R2014a platform, American MathWorks company.

## Results

**FIGURE 3:**
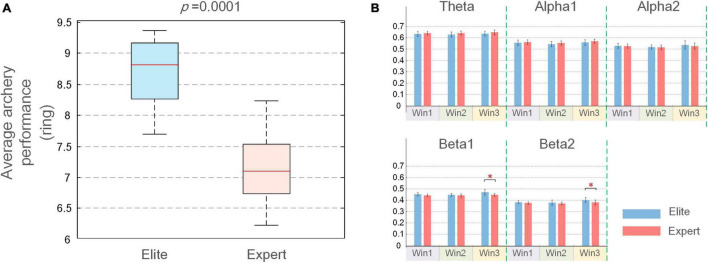
**(A)** Mean and *SD* of the average performance of elite and expert archers; **(B)** mean and *SD* of average weighted phase lag index (WPLI) coupling values for elite and expert groups over three-time windows. In the figure, the longitudinal axis is the functional coupling strength, the error bar represents the *SD*, and the asterisk at the top represents the difference in the average coupling value between the two groups (*p < 0.05).

Before examining the functional coupling and topological characteristics of EEG, we first studied the differences in archery behavior indicators with respect to IAF between the two archer groups. We conducted the K–S test on the IAF of each subject in the two groups and found that IAF did not follow a normal distribution (*p* < 0.05); further, Wilcoxon signed-rank test found no significant difference in IAF between the two groups (*p* > 0.05). According to the archery performance indicators, as shown in Figure 3A, the average ring value of the elite group was 8.7 (±0.56) and that of the expert group was 6.8 (±1.44). The results of the K–S test showed that neither group’s archery performances were normally distributed (*p* > 0.05), and the Wilcoxon signed-rank test result of *p* < 0.01 was used, which indicated that the average score of the elite group was significantly higher than that of the expert group.

### Differences in Functional Coupling Between Elite and Expert

The average WPLI connection strength on the five frequency bands (theta, alpha1, alpha2, beta1, and beta2) and three windows (Win1 to Win3) was analyzed in the elite and expert groups. The most suitable frequency band and time window were determined by comparing the significant differences in connection strength between the two groups. As shown in Figure 3B, the average coupling value of the two archer groups only showed a significant difference in beta1 and beta2 bands of the Win3 window. Meanwhile, in order to ensure the accuracy of experimental results, we also calculated the average connection strength of all subjects on each channel and tested it by the Wilcoxon rank-sum test. The *p*-values in the statistical results were listed in Supplementary Appendix 3. By comparing the number of significant nodes in all-time windows and frequency bands, the largest significant difference between beta1 and beta2 bands in the Win3 window was confirmed again. Therefore, beta1 and beta2 bands in the Win3 window were considered the analysis time window and analyzed frequency band, respectively, in this experiment, and EEG characteristics in this frequency band were mainly studied.

**FIGURE 4:**
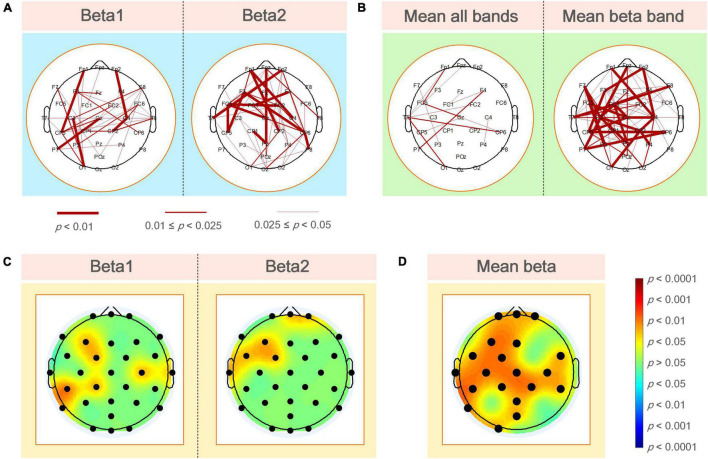
**(A)** WPLI couplings of the elite and expert groups had a significantly different connection in the analyzed frequency bands. Nodes represent electrode positions, and connections between nodes represent statistically significant differences between the two groups (*p* < 0.05). The red connections indicate that the elite archer is stronger than the expert archer, and the thicker lines indicate that the connection is more significant; **(B)** the elite and expert groups had significantly different connections in the average WPLI of all frequencies and analyzed frequencies; **(C)** the average WPLI couplings of each node in the elite and expert groups showed significant differences in the analyzed frequency bands of the brain topographic map, with black nodes representing electrode positions; the darker the color in the map, the more significant the difference; **(D)** the average WPLI connections of the elite and expert groups’ nodes have a significant difference in the average beta band connection value of the total connection strength difference graph. The black nodes are the electrode positions with a significant difference in connection.

Figure 4A shows the statistical test results of a significant difference in functional coupling strength between elite and expert archers in the analyzed frequency band (beta1 and beta2) of the Win3 window. The line between nodes represents the WPLI connection with significant difference between the two archer groups (*p* < 0.05); the thickness of the connection between nodes indicates the degree of significance; the higher the degree of significance, the thicker the connection line, and vice versa. Below the figure is the relationship between the thickness of the connection and the *p*-value of significance. No connection between nodes indicates no significant difference between the two archer groups’ connections. Furthermore, red indicates that the connection of elite archers is stronger than that of expert archers, and blue indicates the opposite. However, as per the statistical results, no blue connection appears in the figure. The results showed that the elite group has higher connection strength in beta1 and beta2 bands than the expert group. In the beta1 band, the difference in functional coupling among nodes Fp1–CP5, Fp2–C4, CP2–C4, C3–O1, and Cz–P7 was the most significant. Beta2 band showed more significantly different connections (*p* < 0.01), which were mainly concentrated in the prefrontal, central, left temporal, and frontal regions, and the most significant differences are found in nodes (*p* < 0.01), such as nodes Fpz, Fp1, Fp2, Fz, F3, FC1, FC2, FC5, T7, Cz, CP5, CP6, and Pz.

As shown in Figure 4B, the left and right sides of the two archer groups showed significant differences in the average connection strength (i.e., the average of all frequencies and average of beta frequencies) obtained by the WPLI connection matrix of all frequencies in the Win3 window (theta, alpha1, alpha2, beta1, and beta2) and the analyzed frequencies after the average of statistical test results of frequency dimensions. Our results also showed more robust functional connectivity in the elite group, and the difference between the two groups on the average beta band was more significant than the average band.

Figure 4C shows the significant coupling differences in different regions of the brain were obtained by the difference test for the average connection strength of the two groups. The regions with color in the brain topographic map indicate the regions with significant differences, with red indicating that the elite group had higher connectivity values than the expert group and blue indicating the reverse. For example, in the beta1 band, there were significant differences in the left frontal (F3: *p* < 0.05), left central (FC1, CP1 and CP5: *p* < 0.05), left parietal (P8: *p* < 0.05), right central (C4: *p* < 0.05), and the right temporal regions. In contrast, in the beta2 band, the differences in average connection strength were mainly concentrated in the prefrontal (Fpz and Fp2: *p* < 0.05), left frontal (F3: *p* < 0.05), left central (CP1 and CP5: *p* < 0.05), and left temporal regions.

By determining the locations of the brain regions with significant differences in different average frequency bands between the two archer groups, we could further analyze the differences in functional coupling characteristics as a whole. Figure 4D shows the different brain topographic maps of the average analyzed frequency band (i.e., the average beta frequency band) obtained by the WPLI brain network matrix of the two archer groups after the average frequency band dimension and the different test, showing the node locations with significant differences in the brain network coupling values of the two archer groups. The color indicator bar on the right represents a statistically significant difference in coupling values between the two archer groups. Based on the results, we found that significant differences between the elite and expert groups were mainly in the prefrontal (Fpz, Fp1, and Fp2: *p* < 0.05), left frontal (F3: *p* < 0.05), central frontal (Fz: *p* < 0.05), central (Cz: *p* < 0.05), left central (FC1, FC5, C3, CP1, and CP5: *p* < 0.05), right central (FC6, FC2, C4, CP2, and CP6: *p* < 0.05), central parietal (Pz: *p* < 0.05), left parietal (P7: *p* < 0.05), parieto-occipital (POz: *p* < 0.05), and left occipital (O1: *p* < 0.05) regions.

**FIGURE 5:**
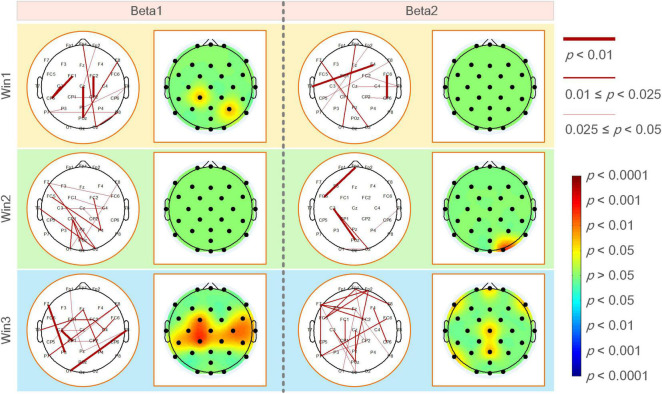
The functional coupling change rate (ErWPLI) of the elite and expert groups had significantly different connections and nodes in analyzed frequency bands within three-time windows, the black nodes in the brain topographic map represented the electrodeposition. The red nodes indicated that the coupling change rate of the elite group was higher than that of the expert group. The darker the color, the more significant the difference.

Figure 5 shows the functional coupling change rate (ErWPLI) of the elite and expert groups in the analyzed frequency bands within the three-time windows relative to the baseline (the functional coupling value of the two groups of archery athletes −4 to −3 s before shooting), and the statistically significant connections and nodes were obtained after the difference test. According to the results, as the shooting time approached, there were several wires and nodes with significant differences in ErWPLI between the two archer groups, which also indicates that the Win3 window is the most suitable window for studying the connection differences between the two archer groups. In the Win3 window, the difference in the beta1 band coupling values was the most significant at F7-P3, C3-CP1, and T8-O1 nodes (*p* < 0.01). In contrast, the difference in the beta2 band was statistically significant in the prefrontal (Fpz: *p* < 0.05), left and right frontal (F7 and F8: *p* < 0.05), central, central parietal, and left parietal (P7: *p* < 0.05) regions.

### Differences in Brain Network Topology Characteristics Between Elite and Expert

**FIGURE 6:**
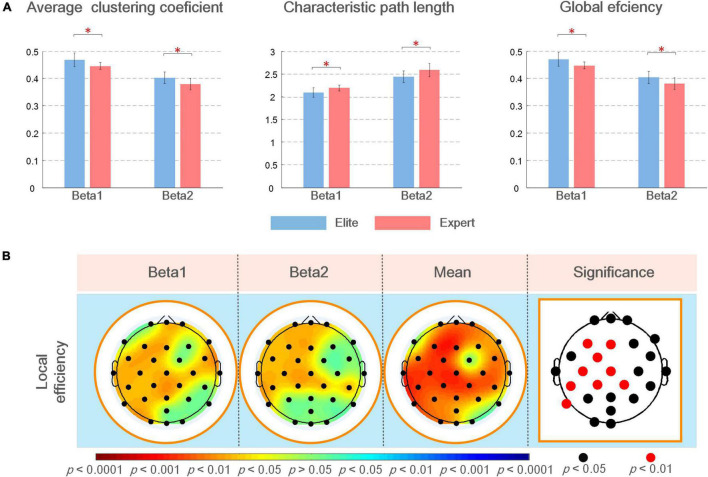
**(A)** Mean and *SD* of global topological characteristics of brain networks in the analyzed frequency bands of the elite and expert groups, and the asterisk at the top represents the difference in the topological characteristics between the two groups (*p < 0.05); **(B)** statistical results of the different analysis of local brain network topology characteristics between elite and expert groups in different analyzed frequency bands. In the figure, from left to the right are the brain topographic maps of connection differences in beta1, beta2, and mean beta band and the nodes with significant differences in statistical results (i.e., electrode positions). In the brain map, red indicates that the elite group has higher eigenvalues than the expert group, blue indicates the reverse; the darker the color, the more significant the difference. In the block diagram, the black/red nodes indicate the significance level (*p* < 0.05/*p* < 0.01).

Figure 6A shows the average value, *SD* of the global topological characteristics of the brain network in the beta1 and beta2 bands in the Win3 window of the elite and expert groups, and the results of the different tests of the two groups of global topologies. As shown in the figure, in the analyzed frequency bands, the average clustering coefficient and global efficiency of the elite and expert groups showed that the eigenvalue of the beta1 frequency band was greater than that of the beta2 frequency band, and the eigenvalue of the elite group was greater than that of the expert group. In contrast, the characteristic path length shows the opposite result. Thus, the global topological characteristics of the two groups showed significant differences in each analyzed frequency band.

In the difference test of local topological characteristics, we only found a significant difference in local efficiency, with no significant difference in eigenvector centrality and average shortest path length. Therefore, in this article, the results of these two parameters are not demonstrated in any figure. As shown in Figure 6B, local efficiency was better in the elite group than in the expert group. In the analyzed frequency bands, the difference in local efficiency between the two groups was the most significant in the left frontal, frontal, and central regions. The significant difference in beta1 frequency band was also noted in the right frontal (F8: *p* < 0.05), right central (FC6, C4, and CP2: *p* < 0.05), right temporal, left parietal, and left occipital regions; further, a significant difference in beta2 frequency band in the right central region (CP2 and CP6: *p* < 0.05) and left parietal (P7: *p* < 0.05) regions was noted. According to the different results in the average beta frequency band, the local efficiency of the two groups was significant (*p* < 0.05) in most regions of the whole brain except F7, FC2, P8, and O2 nodes. Among them, the nodes F3, FC1, C3, CP1, CP5, CP2, and P7 all showed the most significant differences (*p* < 0.01).

### The Correlation Between Brain Network Characteristics and Archery Performance in Elite and Expert

**FIGURE 7:**
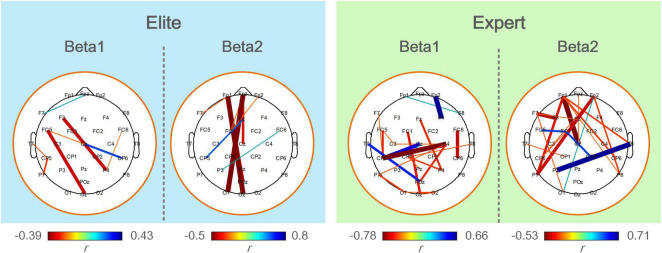
The correlation analysis between functional coupling strength of the elite and expert groups and archery performance in the analyzed frequency bands. Colored connections represent functional couplings that have a significant correlation (*p* < 0.05) with archery performance, red represents a positive correlation, blue represents a negative correlation, and the color bar represents the difference in a correlation coefficient (*r*). The darker the color and the thicker the line, the greater the correlation coefficient and the higher the degree of significance.

Figure 7 shows the calculated results of the correlation between the functional coupling strength of beta1 and beta2 bands and archery performance of the two archer groups in the Win3 window. The expert group showed significantly correlated connections (*p* < 0.05) in the left central, left parietal, and left temporal regions of the beta1 band and the prefrontal, central, and right temporal regions of the beta2 band. Connections of F3-P4, FC5-Oz, and Cz-CP6 in the beta1 band showed a significant positive correlation in the elite group (*p* < 0.01), with the connections between prefrontal and occipital regions (Fpz-O1 and Fp1-OZ) in the beta2 band showing the most significant correlation (*p* < 0.01).

**FIGURE 8:**
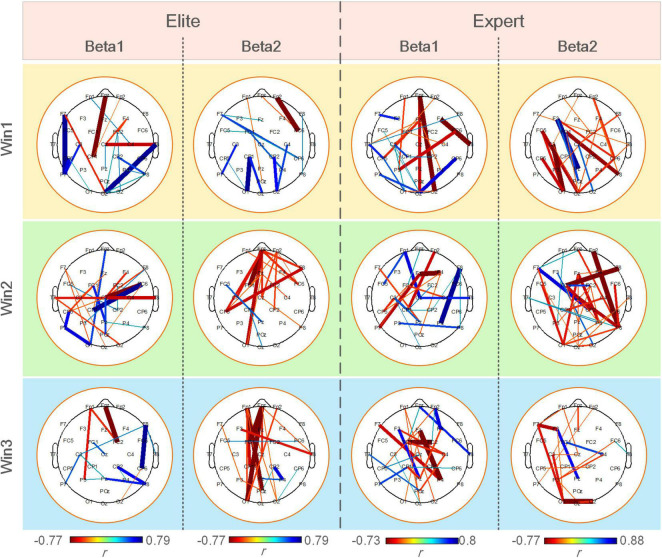
Significantly correlated connections (*p* < 0.05) between the ErWPLI of the elite and expert groups in three-time windows and archery performance.

Figure 8 shows a statistically significant correlation between ErWPLI of the elite and expert groups and archery performance in three-time windows. The figure indicated more connection with a significant positive correlation (*p* < 0.05), and the changes in the WPLI coupling of the expert group were more closely correlated to archery performance. The changes in WPLI coupling of the elite group in the beta1 band and those of the expert group in the beta2 band showed extremely less correlation with archery performance as the shooting time approaches.

According to the results of the correlation test between the topological characteristics of brain network and archery performance of the two archer groups in the Win3 window, we only found a significant correlation with archery performance in the local topological characteristics of the eigenvector centrality and average shortest path length. In contrast, none of the global topological characteristics showed a significant correlation with archery performance.

As shown in Table 1, in the eigenvector centrality, elite archers showed significant correlation at F3 and P4 nodes in the beta1 frequency band, and expert archers showed significant correlation at Fp1 and FC6 nodes in the beta2 frequency band. Furthermore, although elite archers did not show a significant correlation between nodes in the average shortest path length, expert archers showed a significant correlation at the nodes of the left temporal and left central regions in the beta1 frequency band.

**TABLE 1:** Significantly correlated nodes (*p* < 0.05) between the local characteristics of the elite and expert groups with archery performance.

Local topological characteristic	Group	Frequency band	EEG channel	*r*	*p*	Brain region
Eigenvector centrality	Elite	beta1	F3	0.577	0.031	Left frontal
			P4	0.537	0.048	Right parietal
	Expert	beta2	Fp1	0.572	0.033	Left prefrontal
			FC6	−0.539	0.047	Right central
Average shortest path length	Expert	beta1	T7	−0.552	0.041	left temporal
		beta2	CP1	−0.559	0.038	Left central

## Discussion

The main aim of this study was to explore the changes in neural activity in cortical areas involved in archery preparation with different skill levels, as well as the physiological implications of these differences in highly trained athletes. Therefore, we performed a detailed analysis and comparison of the brain network characteristics of the two archer groups in accordance with the specific physiological features of different frequency bands and brain regions in the following paragraph.

### The Most Significant Difference Between Elite and Expert Archers in the Time Window and Frequency Band

First, the EEG frequency band and time window most suitable for analyzing archery preparation were determined. Previous studies on the optimal timing for analysis during firing have suggested that EEG characteristics become more prominent as the shooting time approaches (Hatfield et al., 1984; Gong et al., 2018). By comparing the average WPLI connection strength differences between the elite and expert groups in all observed frequency bands between Win1 and Win3 windows, we found that there were significant differences in frequency bands in the Win3 window. These observations are consistent with the conclusions of previous studies (Kerick et al., 2001; Del Percio and Babiloni, 2009; Gong et al., 2018). We also analyzed the differences in the functional coupling change rate of the two archer groups in three stages and found further different results in the Win3 window. These results indicate that the coupling value and neural changes in the brain of professional archers at different levels showed the most obvious difference at the nearest moment of shooting, and we also speculate that this moment is the most appropriate moment to analyze the neural activity of the brain of professional archers.

The characteristics of brain networks in different frequency bands reflect different physiological aspects of the brain. Previous studies have suggested that theta rhythm usually occurs during sleepiness and negative emotions (Fisch, 1991); alpha1 rhythm is related to the attention and arousal of the whole brain, whereas alpha2 rhythm reflects specific nervous system oscillations and is related to sensorimotor or semantic memory (Klimesch, 1999); beta rhythm is related to motor behavior and active problem solving and usually occurs when an individual is nervous, alert, and excited (Wang and Hsieh, 2013), it also plays a vital role in cognitive processing that requires attention (Ray and Cole, 1985; Gola et al., 2013). Archers in the elite group showed higher connection strength in beta rhythm, indicating a higher degree of brain activation. Thus, we speculated that archers in the elite group perceived and controlled specific movements obviously when completing specific movements. Previous studies have demonstrated that the beta band is more suitable for analyzing aiming behavior than other bands (Vinck et al., 2011; Gong et al., 2018); therefore, it is reasonable to assume that beta1 and beta2 bands are ideal bands to study the differences in the ability of skilled archers; further, we speculated that the brain activity differences in this frequency band with the approaching of the shooting time of archers are an essential reference for determining whether they have the potential to become top archers.

### Analysis of Functional Coupling Differences Between Elite and Expert Archers

Functional coupling characteristics reflect the level of consciousness, the ability of the brain to integrate information, and the development of cognitive functions by studying the information interaction between two different cortical regions. By comparing the average WPLI connection strength of the two archer groups in the three-time windows of Figure 3B, although no significance was shown in theta, alpha1, and alpha2 frequency bands, we found that the average coupling values of the expert archers tend to be higher than those of elite archers in the theta and alpha1 bands, whereas the opposite results were found in the alpha2, beta1, and beta2 bands. This suggests that the brain networks of elite archers are more closely related to cortical regions in the frequency bands associated with archery performance and communicate information more effectively during aiming. Archers should adjust muscle contraction, breath control, and visual aim and should always maintain a high level of attention during aiming, which involves several physiological processes (Lee, 2009; Kim et al., 2019; Sarro et al., 2021). Gallicchio et al. (2016) mentioned that successful shooting performance requires inhibition of irrelevant cognitive processes and enhancement of relevant cognitive processes. We also found that expert archers more precisely utilize neural activity in frequency bands associated with archery behavior during aiming than elite shooters. From the perspective of neural efficiency, elite archers have a better ability to mobilize the neural mechanisms related to archery and have stronger neural efficiency than expert archers.

According to the analysis of connection differences in the frequency band, the electrodes with the most significant connection differences in the beta1 band were mainly distributed in the prefrontal, the left and right central, the left parietal, and the right temporal regions. Conversely, the differences in the beta2 band are concentrated in the prefrontal, left frontal, and left temporal regions. By comparing the functions of each brain area and the corresponding physiological features of the beta1 and beta2 bands, we believe that the experimental results are likely to reflect that the two archer groups have good spatial awareness, and elite archers were better at target recognition and concentration when aiming. Furthermore, the differences were speculated to be related to the psychological state of the archers in the experiment. Although several studies have revealed the physiological features corresponding to different brain regions from the perspective of power changes, the functional connectivity among different brain regions in EEG cannot be equal to power changes, i.e., it cannot represent the synchronous activity of numerous neurons in the brain region but only indicates the phase-locking relationship among neural activities of different brain regions (Kiroi and Aslanyan, 2006). Therefore, the significant difference in functional connectivity between the two groups suggests that closer information exchange among these brain regions during aiming may be the key to elite archers achieving better archery performance than expert archers.

Excellent psychological quality is the prerequisite for archers to achieve excellent archery performance. Increased energy in the beta band leads to brain arousal, which promotes the brain’s processing of motor information (Ebersole and Pedley, 2003). The results showed that the average WPLI coupling values in the average beta band were significantly higher in the elite group than in the expert group, suggesting that the elite group’s brain arousal or sensorimotor information processing ability was greater. It is generally believed that the cognitive processing ability of athletes reflects the level of their skills and determines the outcome of the competition. Because the beta rhythm is related to cognitive processing, the above results also reflect the stronger cognitive processing ability of elite archers. Additionally, Abrams et al. (2013) believed that effective information communication among specific functional brain regions is necessary for good cognitive processing, confirming this article’s conclusion.

In terms of the coupling change rate of functional connection, by analyzing the connection and node differences in the connection strength of the two groups compared with the baseline, we found that the ErWPLI of the elite group was higher in the Win3 window and the difference in functional coupling changes in the beta1 band was the most significant in the central and right temporal regions. Conversely, significant differences in the beta2 band are found in the prefrontal, left and right frontal, central, central parietal, and left parietal regions. It is commonly believed that beta1 rhythm plays a role in integrating sensorimotor information (Pfurtscheller et al., 2003; Baker, 2007) and is also associated with increased arousal and attention (Fan et al., 2007; Heinrich et al., 2007). Beta2 rhythm usually occurs after stimuli and is accompanied by subjective emotions of vigilance, excitement, and anxiety. Based on the corresponding physiological features of beta1 and beta2 bands, and the significant frontal, central, right temporal, and left parietal regions corresponding to the functions of the brain in executing the planning, processing fine motor skills, mental cognitive processing, target recognition, and attention (Marzbani et al., 2016), we exploratively believe that elite archers more rapidly enhance the arousal level of these brain regions in Win3 window, allowing the brain to strengthen its ability to recognize targets and focus on mental cognitive processing to process fine motion skills.

### Analysis of Brain Network Topology Differences Between Elite and Expert Archers

To better understand the operation process of the brain network and analyze network complexity from the perspective of functional integration, we adopted the graph theory method to analyze WPLI-based brain network differences between the elite and expert groups. In graph theory, characteristic path length and average clustering coefficient are two measures to indicate functional integration and separation of the functional brain network, respectively (Fair et al., 2007; Stam and Straaten, 2012; Sporns, 2013). Global efficiency can represent the global transmission capacity and information integration efficiency on the network. It is generally believed that the larger the clustering coefficient, the higher the local efficiency of information processing, whereas the smaller the characteristic path length, the higher the global efficiency of information processing. According to the global topological difference, a larger average clustering coefficient of elite archers reflects the higher degree of information aggregation and processing abilities of the brain, whereas smaller characteristic path length means higher overall routing efficiency between the area of the brain and the integration of potential function, suggesting that the local and overall information integration abilities of the brain of elite archers were stronger than those of elite archers. Furthermore, from the perspective of small-world characteristics of the brain network, compared with the expert archers, the elite archers had higher clustering coefficient and lower characteristic path length, which indicates that elite archers have more small-world characteristics, with higher overall organization efficiency of brain network and closer communication of brain information (Gong et al., 2019).

According to the results of the analysis of local characteristics of the brain network, there were significant differences in more nodes of the frequency band of which local efficiency was analyzed between the two archer groups, and the characteristic values of elite archers were stronger than those of expert archers. Local efficiency measures the degree of local information clustering and information exchange efficiency, indicating that the local neural connection of elite archers was more intensive and efficient than that of expert archers. Regarding the aiming stage of archery, Kim et al. (2014) research showed that excellent professional archers have more limited neural activity during the aiming stage than novice archers, and higher local efficiency can help the archery-related regions of the brain to process complex processes, which also strongly supports the results of this experiment. In the average beta band, the nodes with significant differences between the two groups are as follows: frontal region (F3 and Fz) is related to negative working memory, concentration, executive planning, and positive emotions; the left central region (FC1, Cz, C3, CP1, and CP5) is related to attention and mental processing; the right central region (CP2) focuses on integrated processing of dexterity, sensation, and movement and the combined processing of fine motor skills; and the left parietal region (P7) is associated with involvement in complex problems and attention (Buschman and Miller, 2007; Marzbani et al., 2016). From the perspective of the function of the above-mentioned brain regions, it is likely that the two archer groups have significant differences in cognitive processing, working memory, and attention concentration. Furthermore, the elite archers were more efficient in processing information related to physiological processes than expert archers.

In this experiment, we found that the difference in the local efficiency of the left brain was more obvious, with more nodes with extremely significant differences. For this result, we believe that it might be due to the right brain being more closely correlated with shooting performance (Deeny et al., 2003, 2009; Del Percio et al., 2011a). The research subjects of this article were experienced professional archers who have practiced archery skills, so there was little difference in the brain regions correlated to archery behavior. The reason for the difference lies more in the grasp of specific movement details, which also corresponds to the physiological function of the left brain associated with attention to detail (Marzbani et al., 2016).

To sum up, the process of archery aiming was fine activity coordinated by multiple types of brain networks. Compared with top archers, even highly skilled archers with the same long-term training also had a large number of significant differences, and a top striker global and local information processing ability of the brain is stronger. However, it should be emphasized that the results of this experiment in the average shortest path length and the eigenvector centrality only indicate that the differences in these characteristics between the two archer groups are insignificant, rather than being unimportant to the archery process. They are the commonalities in the brain activities of the two archer groups, and they are jointly involved in the neural activities related to archery tasks.

### Correlation Analysis of Brain Network Characteristics and Archery Performance Between Elite and Expert Archers

Based on a study of the differences in EEG characteristics of professional archers, the correlation between the two archer groups and their archery performance was further analyzed. The neural adaptivity hypothesis indicates that high-level athletes had better adaptability and could complete good skill execution under different conditions, but the correlation between brain neural activity and performance was not high (Bertollo et al., 2016). Moreover, our results indicate that the number of connections significantly correlated with archery performance in the Win3 window in elite archers is less than that in the expert archers, which means that the neural adaptivity is stronger and archery performance is more independent on the state of neural activity in elite archers, and they can show good archery performance in any state of the brain.

According to the correlation between the ErWPLI and archery performance, the neural activity of the elite archers in the beta1 band and the expert archers in the beta2 band showed less and less correlation with the advent of the shooting moment. This can also be explained by the neural adaptivity hypothesis, which indicates that both groups of subjects have strong brain regulation ability and mental state before shooting. In addition, from the point of view of the number of relevant connections in Figure 8, compared with the functional coupling strength, the ErWPLI has a more significant correlation with archery performance. This may suggest that the changes in neural coupling are more closely correlated to archery performance and are more suitable as a characteristic to reflect the correlation between archer and archery performance.

In the study of the topological characteristics of the brain network of the two archer groups, only the correlation between the eigenvector centrality and average shortest path length was statistically significant. Because there was no significant difference in these topological characteristics in the experimental results, it is suggested that these significantly correlated brain regions jointly play an essential role when professional archers aim to shoot. The eigenvector centrality reflects the importance of nodes (Sporns et al., 2007), which indicates that these nodes with significant correlation are key nodes during aiming. The results also indicate that these characteristics of elite archers were significantly positively correlated with archery performance in the left frontal and right parietal regions. According to the corresponding physiological features in the relevant brain regions, we believe that elite archers have better spatial awareness and more focused attention and speculate that specifically activating these positively correlated nodes will positively affect archery performance. In the average shortest path length, expert archers are negatively correlated in the left temporal (T7) and left central (CP1) regions. The average shortest path is inversely proportional to the information transmission efficiency in the brain. The shorter the average shortest path length of the archer’s brain, the higher the information transmission efficiency and the better the archery performance. In addition, the CP1 node is also commonly classified as the superior parietal lobe, and the two-stream hypothesis proposes that the dorsal stream (also known as the parietal stream) is involved in spatial awareness and motor guidance, involving somatosensory processing, movement, and association (Mishkin and Ungerleider, 1982). This also seems to suggest that the higher local information transmission efficiency in these brain regions and better spatial awareness can help the archer achieve better archery performance.

### Research Limitations

This was a data-driven study, and the experiment did not preset regions of interest; therefore, this study has a certain scientific and comprehensive value. While exploring the differences, we also found the commonalities and proved the experimental results from physiological and psychological perspectives. Although the analysis has allowed us to understand the differences between elite and expert archers and the characteristics of archery-correlated brain networks, there are still some limitations in this study. First, owing to the limited number of top athletes, although the experiment included a considerable number of subjects, the data of these subjects are still less. This may result in the lack of independence of research objects, making it difficult for us to explain the characteristic values and their differences and draw limited conclusions. For example, in this experiment, no scientific and reasonable explanation has been obtained for the simultaneous occurrence of a significant positive and negative correlation in the functional coupling and the coupling change rate correlated with archery performance. Second, in the part of analysis and discussion, various conclusions of shooting research are cited, which may lead to inaccurate analysis and argument. To investigate the underlying reason, although the process of shooting and archery is similar, the relationship between brain activity and archery behavior process is extremely complex. The shooting process is different from archery; it can employ lying, kneeling, and standing postures, and the subjects in the shooting experiment are mostly experts and novices; the skill of shooters in the expert group did not reach the level of the top athletes in this study. Therefore, we cannot simply extend the physiological significance of rifle shooting to the related areas of brain activity during archery. Finally, although the functional coupling method of WPLI was employed in this study to address the common source problem to some extent, the obtained brain network matrix is not directional; therefore, the method cannot measure the causal relationship between the EEG signals of two brain regions (or electrodes). The next step would be to explore the development direction of EEG characteristics used by athletes at different levels in task-specific states using an effective connection to describe the causal relationship between events to establish a weighted brain network.

## Conclusion

From the perspective of functional brain networks, this study analyzed and compared brain network characteristics between 14 elite and 14 expert archers in the archery preparation stage and the correlation with archery performance. The results showed significant differences between the two groups in beta1 and beta2 bands within the Win3 window. The functional coupling, as well as local and overall information integration efficiency of elite archers, was stronger than that of expert archers in this band; however, it has less correlation with archery performance. This study showed that the information interaction between the specific functional cortex of elite archers was closer, which can more efficiently improve the level of brain arousal and cognitive processing, and speculated that these characteristics make the brain more neurologically adaptable to focus on the control of movement details. These results verify the hypothesis, which can provide a new physiological basis for professional athletes to further improve their skills, as well as a valuable reference for future exploration of neural activities in fine sports related to archery.

## Data Availability Statement

The raw data supporting the conclusions of this article will be made available by the authors, without undue reservation.

## Ethics Statement

The studies involving human participants were reviewed and approved by the Ethics Committee of the Capital Institute of Physical Education. The patients/participants provided their written informed consent to participate in this study.

## Author Contributions

FG and AG collected the experimental data and wrote the original manuscript. YQ analyzed the experiments results. AB and JW designed the experiments and revised the manuscript. CJ and YF provided an important advice and help on key content of the manuscript. All authors contributed to the article and approved the submitted version.

## Conflict of Interest

The authors declare that the research was conducted in the absence of any commercial or financial relationships that could be construed as a potential conflict of interest.

## Publisher’s Note

All claims expressed in this article are solely those of the authors and do not necessarily represent those of their affiliated organizations, or those of the publisher, the editors and the reviewers. Any product that may be evaluated in this article, or claim that may be made by its manufacturer, is not guaranteed or endorsed by the publisher.
