# Supplementary material for: From Expert to Elite? — Research on Top Archer’s EEG Network Topology
Source: Front Hum Neurosci. 2022 Feb 25;16:759330. doi: 10.3389/fnhum.2022.759330 (PMC8916709; doi:10.3389/fnhum.2022.759330)
Supplement: Supplementary file 1 [file Data_Sheet_1.docx]

Appendix 1

Information for athletes of the Beijing archery team

| Subject | Age | Height (cm) | Weight (kg) | Training years | Sport level | Main competitions participated | FITA scores | World Ranking | Archery performance |
| --- | --- | --- | --- | --- | --- | --- | --- | --- | --- |
| Sub1 | 25 | 178.0 | 90.0 | 3.0 | Second level | National Championship | — | — | 7.0 |
| Sub2 | 22 | 178.0 | 76.0 | 6.0 | First level | National Championship | — | — | 7.0 |
| Sub3 | 24 | 182.0 | 78.0 | 4.0 | Second level | National Championship | — | — | 7.4 |
| Sub4 | 21 | 172.0 | 73.0 | 6.0 | First level | National Championship | — | — | 7.3 |
| Sub5 | 26 | 180.0 | 122.0 | 4.0 | Second level | National Championship | — | — | 7.5 |
| Sub6 | 23 | 178.0 | 66.0 | 4.0 | Second level | National Championship | — | — | 8.2 |
| Sub7 | 26 | 178.0 | 78.0 | 8.0 | First level | National Championship | — | — | 6.7 |
| Sub8 | 24 | 172.0 | 88.0 | 4.0 | Second level | National Championship | — | — | 5.2 |
| Sub9 | 23 | 178.0 | 75.0 | 5.0 | First level | National Championship | — | — | 7.2 |
| Sub10 | 22 | 163.5 | 66.0 | 4.0 | Second level | National Championship | — | — | 7.0 |
| Sub11 | 22 | 168.0 | 63.0 | 5.0 | Second level | National Championship | — | — | 2.4 |
| Sub12 | 20 | 173.0 | 67.5 | 2.0 | Second level | National Championship | — | — | 7.5 |
| Sub13 | 19 | 170.0 | 67.0 | 3.0 | Second level | National Championship | — | — | 7.8 |
| Sub14 | 25 | 168.0 | 56.0 | 4.0 | Second level | National Championship | — | — | 8.1 |
| Sub15 | 23 | 175.0 | 67.0 | 3.0 | Second level | National Championship | — | — | 6.2 |

Information for athletes of China national archery team

| Subject | Age | Height (cm) | Weight (kg) | Training years | Sport level | Main competitions participated | FITA scores | World ranking | Archery performance |
| --- | --- | --- | --- | --- | --- | --- | --- | --- | --- |
| Sub1 | 28 | 174.0 | 68.0 | 6.0 | International master sportsman | Rio Olympics | 55.4 | 77 | 8.9 |
| Sub2 | 24 | 164.0 | 63.0 | 11.0 | International master sportsman | Asian Games | 30.3 | 131 | 8.3 |
| Sub3 | 22 | 172.0 | 63.0 | 8.0 | master sportsman | Tokyo Olympics | 62.3 | 60 | 7.7 |
| Sub4 | 22 | 173.5 | 67.0 | 8.0 | International master sportsman | Rio Olympics | 77.7 | 47 | 8.7 |
| Sub5 | 20 | 170.0 | 68.0 | 6.0 | master sportsman | Rio Olympics and Tokyo Olympics | 82.5 | 43 | 8.8 |
| Sub6 | 23 | 168.0 | 63.0 | 9.0 | International master sportsman | World Cup | 147.0 | 12 | 9.1 |
| Sub7 | 22 | 177.0 | 71.0 | 6.0 | International master sportsman | World Cup | 116.5 | 25 | 8.8 |
| Sub8 | 26 | 185.0 | 94.0 | 12.0 | International master sportsman | Tokyo Olympics | 85.9 | 41 | 9.2 |
| Sub9 | 26 | 172.0 | 80.0 | 10.0 | master sportsman | World Cup | 16.2 | 236 | 8.8 |
| Sub10 | 23 | 184.0 | 75.0 | 10.0 | International master sportsman | World Championships | 45.8 | 96 | 9.2 |
| Sub11 | 23 | 172.0 | 70.0 | 9.0 | International master sportsman | World Cup | 35.2 | 131 | 8.3 |
| Sub12 | 24 | 173.0 | 77.0 | 7.0 | master sportsman | World Cup | 14.4 | 255 | 9.4 |
| Sub13 | 22 | 183.0 | 75.0 | 5.0 | International master sportsman | World Championships | 51.3 | 87 | 8.0 |
| Sub14 | 18 | 185.0 | 77.0 | 7.0 | International master sportsman | Tokyo Olympics | 62.3 | 72 | 9.0 |
| Sub15 | 23 | 177.0 | 72.0 | 8.0 | master sportsman | Asian Games | 28.2 | 147 | 9.3 |
| Sub16 | 22 | 184.0 | 75.0 | 7.0 | master sportsman | World Cup | 24.6 | 160 | 7.7 |

Appendix 2

The clustering coefficient is calculated as follows:

$\text{C}_{\text{p}}=\frac{1}{n}\sum_{i\in N} \text{C}_{i}=\frac{1}{n}\sum_{i\in N} \frac{2t_{i}}{k_{i}(k_{i}-1)}$, (3)

where $\text{C}_{\text{p}}$represents the clustering coefficient of the brain network, which is the mean value of clustering coefficients of all nodes, $\text{C}_{i}$is the local clustering coefficient of each node, *n* represents the total number of nodes, $t_{i}$represents the ratio of the sum of weights of actual edges in nodes adjacent to node *i* to the maximum possible weights in neighboring nodes, and $k_{i}$ represents the node degree of this node (Onnela et al., 2005).

The characteristic path length is calculated as follows:

$\text{L}_{\text{p}}=\frac{1}{n}\sum_{i\in N} \frac{\sum_{j\in N,j\neq i} d_{ij}}{n-1}$, (4)

where $\text{L}_{\text{p}}$ represents the length of the characteristic path of the brain network and is the average of the length of the characteristic path among all node pairs. In the weight matrix, the connection length of edges is the reciprocal of the connection strength, and the characteristic path of each pair of nodes is the path with the shortest total length from node *i* to node *j* (Watts et al., 1999).

Global efficiency is calculated as follows:

$\text{E}_{\text{g}}=\frac{1}{n}\sum_{i\in N} \frac{\sum_{j\in N,j\neq i} (d_{ij})^{-1}}{n-1}$, (5)

where $\text{E}_{\text{g}}$ represents the global efficiency of the network and is the average of the inverse of the shortest path of all node pairs (Latora et al., 2001). It can be seen from the above equation that the global efficiency is inversely proportional to the shortest path of the node.

The eigenvector centrality is calculated as follows:

$\text{V}_{i}=u\sum_{j=1}^{n} a_{ij}x_{j},\text{ Ax}=\lambda x\text{, x}=\frac{1}{\lambda}\text{Ax}$, (6)

where $\text{V}_{i}$ represents the eigenvector centrality of a node, *A* represents the adjacency matrix of WPLI, *λ* represents the maximum eigenvalue, *x* represents the eigenvector, and *u* represents 1/*λ*.

The average shortest path length is calculated as follows:

$D_{i}=\frac{1}{N-1}\sum_{a_{uv}\in\text{g}_{i\leftrightarrow j}}^{N} a_{uv}$, (7)

where $D_{i}$ represents the average shortest path length of node *i* and $\text{g}_{i\leftrightarrow j}$ represents the shortest distance between node *i* and node *j* in the network; if nodes *i* and *j* are not connected, then $\text{g}_{i\leftrightarrow j}$=$\infty$ (Rubinov et al., 2010).

The local efficiency is calculated as follows:

$\text{E}_{l}\text{=}\frac{1}{2}\sum_{\text{i}\in\text{N}} \text{E}_{\text{i}}\text{=}\frac{\sum_{\text{j,h}\in\text{N,j¹i}} \left(\text{a}_{\text{ij}}\text{a}_{\text{jh}}\left[\text{d}_{\text{jh}}^{\text{w}}\left(\text{N}_{\text{i}}\right)\right]^{\text{-1}}\right)^{\frac{1}{3}}}{\text{k}_{\text{i}}\left(\text{k}_{\text{i}}\text{-1}\right)}$,  (8)

where $\text{E}_{l}$ represents the average local efficiency of the network, $\text{E}_{\text{i}}$ is the local efficiency of node *i,* and $\text{d}_{\text{jh}}^{\text{w}}\left(\text{N}_{\text{i}}\right)$ represents the shortest path length from node *j* to node *h* and includes only node *i* (Latora et al., 2001).

Appendix 3

The *p*-values of average channel functional coupling strength between elite and expert groups were obtained by the Wilcoxon signed-rank test in the Win1 time window.

|  | channel | theta | alpha1 | alpha2 | beta1 | beta2 |
| --- | --- | --- | --- | --- | --- | --- |
| 1 | Fp1 | 0.3575 | 0.7148 | 0.6698 | 0.9032 | 0.4263 |
| 2 | Fpz | 0.6698 | 0.2676 | 1.0000 | 0.3575 | 1.0000 |
| 3 | Fp2 | 0.1189 | 0.7148 | 0.6257 | 0.1040 | 0.5016 |
| 4 | F7 | 0.1353 | 0.4263 | 0.8552 | 0.6698 | 0.5416 |
| 5 | F3 | 0.8077 | 0.4631 | 0.4263 | 0.9515 | 0.1531 |
| 6 | Fz | 0.8552 | 0.2676 | 0.5416 | 0.3575 | 0.3575 |
| 7 | F4 | 0.3258 | 0.2412 | 0.7148 | 0.3910 | 0.9515 |
| 8 | F8 | 0.0906 | 0.3575 | 0.1531 | 0.8077 | 0.4631 |
| 9 | FC5 | 0.1189 | 0.9032 | 0.6698 | 0.6698 | 1.0000 |
| 10 | FC1 | 0.8077 | 0.8552 | 0.2412 | 1.0000 | 0.0676 |
| 11 | FC2 | 0.9515 | 0.7609 | 0.5416 | 0.0353* | 0.4263 |
| 12 | FC6 | 0.5830 | 0.6698 | 0.9032 | 0.8077 | 0.3575 |
| 13 | T7 | 0.2412 | 0.8077 | 0.3258 | 0.9032 | 0.0906 |
| 14 | C3 | 0.8077 | 0.6698 | 0.2412 | 0.2412 | 0.5016 |
| 15 | Cz | 0.2676 | 1.0000 | 0.5016 | 0.0166* | 1.0000 |
| 16 | C4 | 0.2412 | 0.9032 | 0.8552 | 0.2958 | 0.1353 |
| 17 | T8 | 0.1531 | 0.5016 | 0.7609 | 0.4263 | 0.2412 |
| 18 | CP5 | 0.8077 | 0.8077 | 0.1531 | 0.0295* | 0.0906 |
| 19 | CP1 | 0.8552 | 0.3575 | 1.0000 | 0.2412 | 0.2412 |
| 20 | CP2 | 0.0906 | 1.0000 | 0.8077 | 0.1726 | 0.0419 |
| 21 | CP6 | 0.4631 | 0.8077 | 0.2958 | 0.2676 | 0.0134* |
| 22 | P7 | 0.8552 | 0.1937 | 0.4263 | 0.0494* | 0.0906 |
| 23 | P3 | 0.6257 | 0.1353 | 0.3258 | 0.3910 | 0.8077 |
| 24 | Pz | 0.6257 | 0.2958 | 0.7148 | 0.2676 | 0.3575 |
| 25 | P4 | 0.9032 | 0.4263 | 0.6257 | 0.0166* | 0.3910 |
| 26 | P8 | 0.0785 | 0.5416 | 0.8077 | 0.2412 | 0.8552 |
| 27 | POz | 0.8077 | 0.3258 | 0.7609 | 0.0580 | 1.0000 |
| 28 | O1 | 0.5016 | 0.7609 | 0.5830 | 0.7609 | 0.5830 |
| 29 | Oz | 0.3575 | 0.8077 | 0.7148 | 0.2166 | 0.5416 |
| 30 | O2 | 0.4263 | 0.9515 | 0.1040 | 0.1040 | 0.9515 |

*Represent the significant difference of characteristics between two groups (*p* < .05).

The *p*-values of average channel functional coupling strength between elite and expert groups were obtained by the Wilcoxon signed-rank test in the Win2 time window.

|  | channel | theta | alpha1 | alpha2 | beta1 | beta2 |
| --- | --- | --- | --- | --- | --- | --- |
| 1 | Fp1 | 0.7609 | 0.0353* | 0.5016 | 0.3258 | 0.2412 |
| 2 | Fpz | 0.6257 | 0.0166* | 0.7148 | 0.5830 | 0.0785 |
| 3 | Fp2 | 0.8077 | 0.0203* | 0.1531 | 0.7609 | 0.2412 |
| 4 | F7 | 0.5416 | 1.0000 | 0.5416 | 0.0676 | 0.5416 |
| 5 | F3 | 0.5016 | 0.3910 | 0.5830 | 0.1189 | 1.0000 |
| 6 | Fz | 0.4631 | 0.3258 | 0.5830 | 0.3910 | 0.7148 |
| 7 | F4 | 0.4631 | 0.0906 | 0.9515 | 0.9032 | 0.1353 |
| 8 | F8 | 0.0017* | 0.8552 | 0.4631 | 0.5016 | 0.6698 |
| 9 | FC5 | 1.0000 | 1.0000 | 0.5830 | 0.5416 | 0.4631 |
| 10 | FC1 | 0.1353 | 0.3258 | 0.3575 | 0.7148 | 0.4263 |
| 11 | FC2 | 0.2958 | 0.0676 | 0.6257 | 0.1353 | 0.7609 |
| 12 | FC6 | 0.2676 | 0.1353 | 0.9032 | 0.3575 | 0.8077 |
| 13 | T7 | 0.5016 | 0.9515 | 0.3258 | 0.6257 | 0.5016 |
| 14 | C3 | 0.0676 | 0.9032 | 0.7148 | 0.2958 | 0.0676 |
| 15 | Cz | 0.3910 | 0.2166 | 0.6257 | 0.3575 | 0.6257 |
| 16 | C4 | 0.5830 | 0.1353 | 0.8552 | 0.0676 | 0.5016 |
| 17 | T8 | 0.0494* | 0.5416 | 1.0000 | 1.0000 | 0.0785 |
| 18 | CP5 | 0.0166* | 0.8552 | 0.6698 | 0.7609 | 0.8077 |
| 19 | CP1 | 0.0785 | 0.8077 | 0.7148 | 0.6698 | 0.3258 |
| 20 | CP2 | 0.1531 | 0.0906 | 0.3258 | 0.1937 | 0.7148 |
| 21 | CP6 | 0.0906 | 0.4263 | 0.5416 | 0.8077 | 0.5016 |
| 22 | P7 | 0.1040 | 0.5830 | 0.4631 | 0.3575 | 0.2166 |
| 23 | P3 | 0.1353 | 0.5416 | 0.3575 | 0.4263 | 0.1937 |
| 24 | Pz | 0.2676 | 0.3258 | 0.6698 | 0.0906 | 0.7148 |
| 25 | P4 | 0.0245* | 0.6257 | 0.7609 | 0.4631 | 0.9032 |
| 26 | P8 | 0.8077 | 0.3575 | 0.7609 | 0.8077 | 0.4263 |
| 27 | POz | 0.0906 | 0.5830 | 0.8077 | 0.5830 | 0.6698 |
| 28 | O1 | 0.2166 | 0.0245* | 0.7609 | 0.7148 | 0.2166 |
| 29 | Oz | 0.0245 | 0.2166 | 0.8077 | 0.0419* | 0.3258 |
| 30 | O2 | 0.3258 | 0.5830 | 0.5416 | 0.1353 | 0.0906 |

*Represent the significant difference of characteristics between two groups (*p* < 0.05).

The *p* values of average channel functional coupling strength between elite and expert groups were obtained by the Wilcoxon signed-rank test in the Win3 window.

|  | channel | theta | alpha1 | alpha2 | beta1 | beta2 |
| --- | --- | --- | --- | --- | --- | --- |
| 1 | Fp1 | 0.1937 | 0.5830 | 0.4263 | 0.0295* | 0.0353* |
| 2 | Fpz | 0.9032 | 0.6698 | 0.5416 | 0.1189 | 0.0052* |
| 3 | Fp2 | 0.0353* | 0.6698 | 0.7148 | 0.0580* | 0.0067* |
| 4 | F7 | 0.5416 | 0.4263 | 0.3910 | 0.8552 | 0.0245* |
| 5 | F3 | 0.0067* | 0.0107* | 0.7148 | 0.0052* | 0.0067* |
| 6 | Fz | 0.2958 | 0.1189 | 0.5016 | 0.0353* | 0.0906 |
| 7 | F4 | 0.4263 | 0.4631 | 0.0494* | 0.1937 | 0.2958 |
| 8 | F8 | 0.0419* | 0.1937 | 0.8077 | 0.0676 | 0.4631 |
| 9 | FC5 | 0.9032 | 0.3575 | 0.4631 | 0.0295* | 0.0107* |
| 10 | FC1 | 0.0580 | 0.2412 | 0.6257 | 0.0067* | 0.0085 |
| 11 | FC2 | 0.4631 | 0.7609 | 0.2166 | 0.1937 | 0.0906 |
| 12 | FC6 | 0.7609 | 0.3575 | 0.3575 | 0.0245* | 0.1353 |
| 13 | T7 | 0.9515 | 0.6257 | 0.1726 | 0.0580 | 0.0085 |
| 14 | C3 | 0.2412 | 0.8077 | 0.4631 | 0.0134* | 0.0245* |
| 15 | Cz | 0.0906 | 0.5416 | 0.4631 | 0.0134* | 0.0203* |
| 16 | C4 | 0.0353* | 0.2412 | 0.2958 | 0.0085* | 0.1531 |
| 17 | T8 | 0.2958 | 0.0785 | 0.9032 | 0.0067* | 0.3575 |
| 18 | CP5 | 0.1726 | 0.4631 | 0.5830 | 0.0002* | 0.0295* |
| 19 | CP1 | 0.1937 | 0.3910 | 0.8077 | 0.0067* | 0.0295* |
| 20 | CP2 | 0.1726 | 0.1726 | 0.8552 | 0.0203* | 0.0245* |
| 21 | CP6 | 0.0134* | 0.2166 | 0.6257 | 0.0906 | 0.0166* |
| 22 | P7 | 0.7148 | 0.1531 | 0.7609 | 0.0107* | 0.0166* |
| 23 | P3 | 0.7148 | 0.9032 | 0.6698 | 0.0166* | 0.3575 |
| 24 | Pz | 0.1189 | 0.8077 | 0.7148 | 0.0906 | 0.0676* |
| 25 | P4 | 0.1937 | 0.1040 | 0.4631 | 0.1726 | 0.0676* |
| 26 | P8 | 0.1726 | 0.3910 | 0.2958 | 0.3910 | 0.0785* |
| 27 | POz | 0.7148 | 0.2676 | 0.1937 | 0.1040 | 0.1040 |
| 28 | O1 | 0.2412 | 0.3258 | 0.1937 | 0.0580 | 0.0494* |
| 29 | Oz | 0.1937 | 0.1353 | 0.7148 | 0.1531 | 0.1937 |
| 30 | O2 | 0.1040 | 0.0906 | 0.9515 | 0.3910 | 0.2412 |

*Represent the significant difference of characteristic between two groups (*p* < .05).
